# Emotional Speech Recognition Based on the Committee of Classifiers

**DOI:** 10.3390/e21100920

**Published:** 2019-09-21

**Authors:** Dorota Kamińska

**Affiliations:** Lodz University of Technology, Institute of Mechatronics and Information Systems, Stefanowskiego 18/22 Str. 90-924 Lodz, Poland; dorota.kaminska@p.lodz.pl

**Keywords:** emotion recognition, speech, committee of classifiers

## Abstract

This article presents the novel method for emotion recognition from speech based on committee of classifiers. Different classification methods were juxtaposed in order to compare several alternative approaches for final voting. The research is conducted on three different types of Polish emotional speech: acted out with the same content, acted out with different content, and spontaneous. A pool of descriptors, commonly utilized for emotional speech recognition, expanded with sets of various perceptual coefficients, is used as input features. This research shows that presented approach improve the performance with respect to a single classifier.

## 1. Introduction

During a conversation people are constantly sending and receiving different nonverbal clues, communicated through speech signal (paralanguage), body movements, facial expressions, and physiological changes. The discrepancy between the words spoken and the interpretation of their actual content relies on nonverbal communication. Emotions are a medium of information regarding feelings of an individual and one’s expected feedback. The ability to recognize the attitude and thoughts from one’s behaviour was the original system of communication prior to spoken language. Understanding the emotional state enhances interaction. Although computers are now a part of human life, the relation between human and machine is far from being natural [1]. Proper identification of emotional state can significantly improve quality of human-computer interfaces. It can be applied for monitoring of psycho-physiological states of individuals e.g., to assess the level of stress or fatigue, forensic data analysis [2], advertisement [3], social robotic [4], video conferencing [5], violence detection [6], animation or synthesis of life-like agents xue2018voice, and many others. Automatic emotion recognition methods utilize various input types i.e., facial expressions [7,8,9], speech [10,11,12], gesture and body language [13,14], physical signals such as electrocardiogram (ECG), electromyography (EMG), electrodermal activity, skin temperature, galvanic resistance, blood volume pulse (BVP), and respiration [15]. Facial expressions have been studied most extensively and about 95% of literature dedicated to this topic focuses on faces as a source, at the expense of other modalities [16]. Speech is one of the most accessible form the above mentioned signals, thus recently it is increasingly significant research direction in emotion recognition. Despite an enormous amount of research, the issue is still far from its satisfactory solution. Analysis of emotional content embedded in speech is an issue that presents multiple difficulties. The main problem is gathering and compiling a database of viable and relevant experimental material. Most available corpora comprise speech samples uttered by professional actors, which are not guaranteed to reflect the real environment with its background noise or overlapping voices. Additionally, individual features of the speaker such as gender, age, origin and social influence can greatly affect universal consistency in emotional speech. The first most important work published before 20th century studying emotions was *The Expression of the Emotions in Man and Animals* by Charles Darwin [17]. Darwin made the first description of the paralanguage conveying emotional states of the speaker. Based on the study of people and different species of animals, he came to the conclusion that there is a direct connection between the modulation of speech signal and the internal state of the individual. He also observed that acoustic signals could trigger emotional reactions of the listener. The theoretical and practical approach suggests that specific paralinguistic cues such as loudness, rate, pitch, pitch contour and formant frequencies contribute to the emotive quality of an utterance. Emotions may cause changes in the way of breathing, phonation or articulation, which are reflected in the speech. For example, states like anger or fear are characterized by fast pace, high values of pitch, wide range of intonation, sudden acceleration of heart rate, increased blood pressure and, in some cases, dry mouth and muscle tremor. The opposite phenomena occur in case of sadness and boredom. Speech becomes slow and monotonous, pitch is reduced without any major changes in intonation. This is caused partially due to activation of the parasympathetic system, relief of cardiac rhythm, blood pressure drop and increased secretion of saliva. Consequently, paralinguistic cues relating to emotion have a huge effect on ultimate meaning of the message [18]. This paper refers to my previous research [19], where the novel method for emotional speech recognition based on committee of classifiers was presented. This method is based on a set of classifiers (nodes) whose individual predictions are combined to make the final decision. Current paper is an extension of the previous approach. I investigated three different type of Polish corpora: acted out, in which the actors repeat the same sentence while expressing different emotional states [20]; acted out, in which the actors repeat several different sentences while expressing different emotional states [2]; spontaneous speech samples collected from live shows and programs such as reality shows [21]. I combined different classification methods as nodes (k-NN, MLP, SL, SMO, Bagging, RC, j48, LMT, NBTree, RF) and juxtaposed several alternative approaches to final voting. This research shows that some of presented approaches improve the performance with respect to a single classifier. A pool of descriptors, commonly utilized for emotional speech recognition, expanded with sets of various perceptual coefficients, is used as input feature vectors. The following list summarises the contributions of this work:

1. This research is carried out on three different types of Polish corpora, which allows the analysis of impact of various type of database on the final result. The classifiers were tested by using mixed sets (corpora-dependent and corpora-independent tests) to verify if acted out database can be used as a training set for application operating in real environment.
2. In comparison to similar research where each classifier is trained with the exact same data, in this paper the whole feature set is divided into subsets before classification process. Despite their similarity (e.g., MFCC and BFCC) different models provide varied results on specific feature subsets, affecting the final assessment. Thus, the most effective model may be selected appropriately for a specific subset (in similar research voting is performed on different classifiers working on the same features). This approach significantly increases accuracy of results, in comparison to related works. Presented algorithm was verified using different voting methods.
3. It presents a thorough analysis of extensive set of features on the recognition of several emotional classes-groups of features are examined separately as well as a whole collection.

The structure of the paper is as following. Next section presents a brief review of works related to speech emotion recognition (SER). Section 3 describes proposed research methodology: relevant corporas of emotional voice, speech signal descriptors and outline of adopted strategy for emotion recognition. Section 4 presents obtained results followed by their discussion. Finally, Section 5 gives the conclusion and future directions of this research.

## 2. Related Works

**Figure 1:**
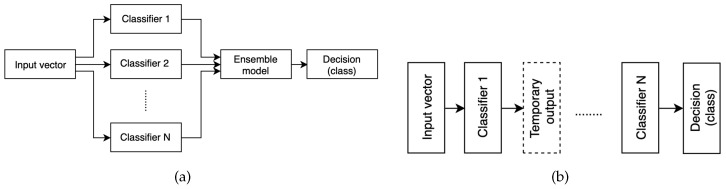
(**a**) Combining the results via simple voting, weighted or highest confidence voting, or other methods. (**b**) Multilevel classification.

Since emotion recognition from speech signal is a pattern recognition problem, standard approach consisting of three processes: feature extraction, feature selection, and classification is used to solve the task. The main research issue is selection of an optimal feature set that efficiently characterizes the emotional content of the utterance. The number of acoustic parameters proven to contain emotional information is still increasing. Generally, the most commonly used features can be divided into three groups: prosodic features (e.g., fundamental frequency, energy, speed of speech) [22], quality characteristics (e.g., formants, brightness) [23] and spectrum characteristics (e.g., mel-frequency cepstral coefficients) [24,25]. The final features vector is based on their statistics such as mean, maximum, minimum, change rate, kurtosis, skewness, zero-crossing rate, variance etc., [26,27]. However, a vector of too many features may give rise to high dimension and redundancy, making the learning process complicated and increasing the likelihood of overfitting [28]. Therefore prior to classification, methods of balancing a numerous features vector, feature selection or extraction are studied to speed up the learning process and minimize the curse of dimensionality problem [29,30]. Emotion classification is generally performed using standard techniques such as SVM [31,32,33], various types of artificial neural networks (NN) [34,35,36,37], different types of the k-NN classifier [19,38] or using Hidden Markov Model (HMM) and its variations [39]. However, it is a complex task with many unresolved issues. Therefore, hybrids and multilevel classifiers [40,41] or ensemble models [42] have been widely used to enhance the performance of single classifiers. Classifying committees (Ensemble, Committee, Multiple Classifier Systems) are based on the principle of *divide and conquer*: they consist of a set of classifiers (nodes) whose individual predictions are combined. A necessary condition for this approach is that member classifiers should have a substantial level of disagreement, i.e., mistakes made by nodes should be independent, regardless of the others. The most commonly used and most intuitive technique consists of several models *C* (Figure 1a) working separately on the same or similar feature set, with their results merged on decision *D* level

This kind of approach was used in [43], where the authors present a multiple classifier system for 5 emotional states (anger, happiness, sadness, boredom and neutral) and task is performed on Mandarin speech. They investigated several classifiers such as k-NN, weighted k-NN, Weighted Average Patterns of Categorical k-NN, Weighted Discrete k-NN and SVM. To combine results, majority voting, minimum misclassification and maximum accuracy methods were compared. The experimental results have shown that classifier combination schemes perform better than the single classifiers with the improvement ranging from 0.9–6.5%. The improvement of the automatic perception of vocal emotion using ensemble methods over traditional classification is shown in [44]. The authors compared two emotional speech data sources: natural, spontaneous emotional speech and acted or portrayed emotional speech to demonstrate the advantages and disadvantages of both. Basing on prosodic features (namely: fundamental frequency, energy, rhythm, and formant frequencies) two ensemble methods (stacked generalisation and unweighted vote) were applied. These techniques shown a modest improvement in prediction accuracy. In [45], the authors analysed the effectiveness of employing five ensemble models such as Bagging, Adaboost, Logitboost, Random Subspace and Random Committee, estimating emotional Arabic speech. The system recognizes happy, angry, and surprise emotion from natural speech samples. The highest improvement in accuracy in relation to the classical approach (19.09%) was obtained by the Boosting technique having the Naïve Bayes Multinomial as the node. Multilevel approach (see Figure 1b) is predicated on splitting the classification process into several consecutive stages. For example in [46] the authors propose a hierarchical classification, which achieves greater accuracy of SER than corresponding classical methods. In the first stage of this algorithm, features vector is used to separate anger and neutral (group 1) from happiness and sadness (group 2). Finally, group 1 is classified into anger and neutrality, and group 2 into happiness and sadness. Similar approach is presented in [47]. First, the emotional states are categorized according to the dimensional model into positive or negative valence and high or low arousal using Gaussian Mixture Model and Support Vector Machines. Final decisions are made inside subsets with fewer categories using spectral representation. Studies were performed using the Berlin Emotional database [48] and the Surrey Audio-Visual Expressed Emotion corpus. In [49], the authors studied the effect of age and gender of the speaker on the effectiveness of emotion recognition system. They proposed a hierarchical classification model to investigate the importance of identifying those features before identifying the emotional label. They compared the performance of four different models and presented the relationship between the age gender and the emotion recognition accuracy. The results proved that using a separate emotion model for each gender and age category gives a higher accuracy compared with using one classifier for all the data. Similarly, in [50], gender is identified on the first level. Next, the dimensional reduction using PCA, LDA and mixed algorithm is performed according to particular gender-set. In [51], the authors underline a fuzzy nature of particular emotional states (e.g., sadness and boredom) and suggest that global classifier cannot obtain effective results. Thus, they proposed a hierarchical approach, which divides the set of utterances into *active* and *passive* on the first level, in order to classify them into emotional categories on the second one. The experiments were conducted on two different corpora: Berlin and DES [52] database. Obtained results outperform those obtained via single classifier.

## 3. Methods

### 3.1. Database

As mentioned in Section 1, for the purpose of this project three different types of Polish datasets were investigated. They will be briefly described below and summarised in Table 1.

#### 3.1.1. MERIP Database

MERIP emotional speech database is a subset of the Multimodal Emotion Recognition in Polish project [20]. The database consists of 560 samples recorded in the rehearsal room of *Teatr Nowy im. Kazimierza Dejmka w Łodzi*. Samples were collected from separate utterances of 16 professional actors/actresses (8 male and 8 female) aged from 25 to 64. The subjects were asked to utter a sentence *Każdy z nas odczuwa emocje na swój sposób* (English translation: *Each of us perceives emotions in a different manner*) while expressing different emotional states in the following order: neutral, sadness, surprise, fear, disgust, anger, and happiness (this set of discrete emotions was based on examination conducted by Ekman in [53]). All emotions were acted out 5 times, without any guidelines or prompts from the researchers. This allowed to gather 80 samples per each emotional state. Audio files were captured using dictaphone Roland R-26 in the form of wav audio files 44,1 kHz, 16 bit, stereo). The samples were evaluated by 12 subjects (6 male and 6 female) who were allowed to listen each sample only once and determine the emotional state. The average emotion recognition rate was 90% (ranging from 84% to 96% for different emotional state).

#### 3.1.2. Polish Emotional Speech Database

The Polish Polish Emotional Speech Database (PESD) [2] was prepared and shared by the Medical Electronics Division, Lodz University of Technology. The database consists of 240 samples recorded in the aula of the Polish National Film Television and Theater School in Lodz. Samples were collected from separate utterances of 8 professional actors/actresses (4 male and 4 female). Each speaker was asked to utter five different sentences (*They have bought a new car today*, *His girlfriend is coming here by plane*, *Johnny was today at the hairdresser’s*, *This lamp is on the desk today* and *I stop to shave from today on*) with six types of emotional load: joy, boredom, fear, anger, sadness, and neutral (no emotion). Audio data was collected in the form of wav audio files (44,1 kHz, 16 bit). The samples were evaluated by 50 subjects through a procedure of classification of 60 randomly generated samples (10 samples per particular emotion). Listeners were asked to classify each utterance into emotional categories. The average emotion recognition rate was 72% (ranging from 60 to 84% for different subjects).

#### 3.1.3. Polish Spontaneous Speech Database

The spontaneous Polish Speech Database (PSSD) [21] consists of 748 samples containing emotional carrier of seven basic states, from the Plutchik’s wheel of emotions [54]: joy, sadness, anger, fear, disgust, surprise, anticipation and neutral. Speech samples were collected from discussions in TV programs, live shows or reality shows and the proportion of speakers’ gender and age was maintained. Each utterance was unique and varied from one-word articulations such as *Yes* or *No*, single words, phrases to short sentences. Occasionally additional sounds such as screaming, squealing, laughing or crying are featured in the corpora. The data was collected in the form of wav audio files of varied quality. The samples were evaluated by 15 male and female volunteers aged from 21 to 58. All listeners were presented random samples that consisted of at least half of each prequalified basic emotions recordings. The evaluators listened to audio samples one by one, each assessment was recorded in the database. Every sample could have been played any number of times before the final decision, but after the classification, it was not possible to return to the recording. Average emotion recognition was 82.66% (ranging from 63% to 93% for different subjects).

To juxtapose these three different databases for the purpose of this project, an equal number of emotional sets was selected, which means that utterances expressing surprise and anticipation were omitted. Additionally, in case of PSSD, the number of samples for emotions has been unified to 80.

### 3.2. Extracted Features

Representation of speech signal in time or frequency domain is too complex to analyze, thus usually high-level statistical features (HLS) are sought to determine its properties. In most cases a large number of HLS features are extracted at the utterance level, which is followed by dimension reduction techniques to obtain a robust representation of the problem. Feature extraction comprises of two different stages. First, a number of low level (LL) features are extracted from short frames. Next, HLS features such as mean, max, min, variance, std, are applied to each of the LLs over the whole utterance, and the results are concatenated into a final feature vector. The role of the HLS is to describe temporal variations and contours of the different LLs during particular speech chunk [55]. Most commonly used LLs, for the purpose of emotional speech recognition, can be divided into two groups: prosodies and spectrum characteristics, both of them described below.

#### 3.2.1. Prosodies

Speech prosodic features are associated with larger units such as syllables, words, phrases, and sentences, thus are considered as supra-segmental information. They represent the perceptual properties of speech, which are commonly used by humans to carry various information [56]. As it has been repeatedly emphasised in the literature, prosodic features such as energy, duration, intonation (*F0* contour) and their derivatives are commonly used as important information sources for describing emotional states.

*F0*, which is the frequency of vocal folds, is inextricably linked with the scale of the human voice, accent and intonation, all of which have a considerable impact on the nature of speech. *F0* does change during utterances and rate of those changes is dependent on the speaker’s intended intonation [22]. For the purpose of this research *F0* was extracted using autocorrelation technique. The analysis window was set to 20 ms with 50% overlap.

Another feature that provides information useful in distinguishing emotions is signal energy, which describes the volume or intensity of speech. For example, some emotional states, like joy or anger, have increased energy levels in comparison to other emotional states.

#### 3.2.2. Spectrum Characteristics

Nowadays, perceptual features are a standard in voice recognition. They are also used in emotional speech analysis. Perceptual approach is based on frequency conversion, corresponding to subjective reception of the human auditory system. For this purpose, the perceptual scales such as Mel or Bark are used. In this paper Mel Frequency Cepstral Coefficients *MFCC* [57], Human Factor Cepstral Coefficients *HFCC* [58], Bark Frequency Cepstral Coefficients *BFCC* [59], Perceptual Linear Prediction *PLP* [60] and Revised Perceptual Linear Prediction *RPLP* [59] coefficients are employed. Additionally, Linear Prediction Coefficients (LPC) [61] were taken into consideration, as they are the most frequently used features for speech recognition. Initially, for all particular perceptual features sets, the number of coefficients has been specified to 12. For all above mentioned LLs sets. HLS such as maximum, minimum, range, mean and standard deviation were determined for all LLs.

Another important feature type, describing properties of vocal tract, are formant frequencies, at which local maxima of the speech signal spectrum envelope occur. They can be utilized to determine the speaker’s identity and the form and content of their utterance [62]. Usually 3 to 5 formants are applied in practice, thus this paper estimates 3 of them and on their basis HLS such as mean, median, standard deviation, maximum and minimum are determined, giving a total of 15 features.

#### 3.2.3. Features Selection

Initially, the number of extracted HLS features amounted to 407. Correlation-based Feature Selection (CFS) algorithm [63] has been applied on the whole set of features as well as on all subsets separately in order to remove redundancy and select descriptors most relevant for analysis.

**Figure 2:**
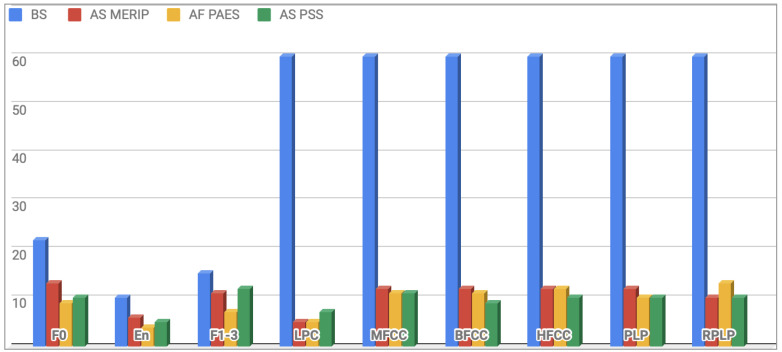
Distribution of features count for particular sets before and after selection process for each database. BS—before selection, AS—after selection.

This procedure resulted in a significant reduction of the feature vector dimension, after CFS the final vectors length was: 93 in case of MERIP, 88 for PESD and 91 for PSSD. Distribution of features before and after the selection process applied on a particular subset is presented in Figure 2. Selected features are presented in Appendix A, Table A1 and Table A2.

### 3.3. Classification Model

**Figure 3:**
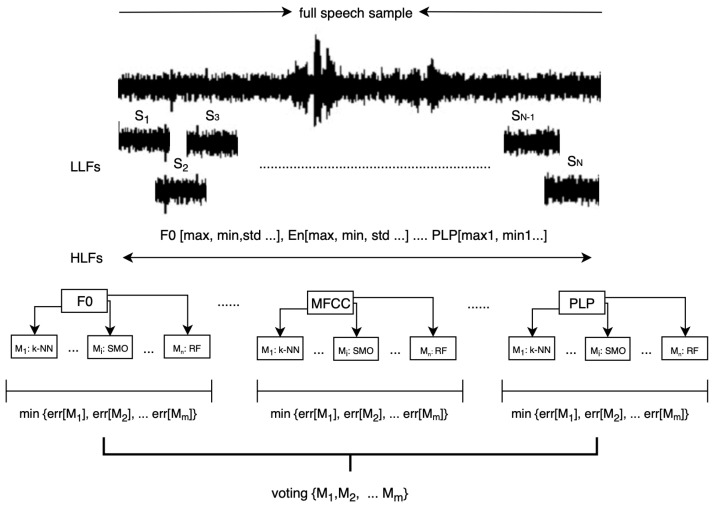
Proposed algorithm for emotion recognition using committee of classifiers.

Proposed algorithm, presented in see Figure 3, starts with division of the HFL feature vector, describing speech samples, into separate sub-vectors of particular group of features (i.e., sub-vector with MFCC coefficients). Each sub-vector is subjected to the selection process, followed by classification using different models *M* (e.g., M1: k-NN, M2: MLP etc.). Subsequently, among the models operating on particular sub-vector, one model with the lowest error rate is selected for further analysis. The error rate is calculated according to Equation (1). Final voting is done among the highest scoring models for particular sub-vectors.

(1) $$err=1-accuracy=1-\frac{\left(\#classified\_correct\right)}{\left(\#classified\_total\right)}=\frac{\left(\#classified\_incorrect\right)}{\left(\#classified\_total\right)}$$

In the basic algorithm, the final decision is made using equal voting. This method does not require additional calculations, only votes of individual models, rendering this process simple and effective. A decision is made collectively, using the following equations:

(2) $$r_{i}=\sum_{j=1}^{m}d_{ji}$$

(3) $$Z=arg\max_{i=1}^{l}\left[r_{i}\right]$$

where: *m*—number of classifiers (models), *l*—number of different classes, $d_{ji}$—decision of *j* classifier for *i* class.

Unequal impact of particular descriptors on the recognition provides the basis for replacing equal with the weighted voting. For each model different weights $w_{1},w_{2},\ldots w_{m}$ are determined, which allows to prioritize more precise models. In this case the Equation (2) is replaced by the following:

(4) $$r_{i}=\sum_{k}^{j=1}d_{ji}w_{j}$$

This approach requires the assessment (or at least comparison) of all models. In this study weights were selected experimentally, based on the error rate of individual classifiers. Appropriate weight $w_{i}$ for individual model was calculated based on the error rate $err_{i}$ according to the following equations.

(5) $$w_{i}=1-err_{i}$$

(6) $$w_{i}=\frac{1}{err_{i}}$$

(7) $$w_{i}={\left(\frac{1}{err_{i}}\right)}^{2}$$

## 4. Results and Discussion

### 4.1. Efficiency of Features Subsets

The verification of efficiency of feature subsets is carried out using several types of classifiers such as k-NN, Multilayer Perceptron (MLP), Simple Logistic (SL), SMO, Bagging, Random Cometee (RC), j48, LMT, NBTree and Random Forest (RF) using Weka [64], with 10-fold cross-validation. This approach allows to evaluate the efficacy of particular features set and determine the most efficient ones. Table 2, Table 3 and Table 4 present the efficiency of above mentioned feature subsets obtained for three independent speech corpora. In the course of research, the parameters for each classifier were identified and selected to achieve the highest recognition results.

It is clearly visible that the best results are achieved for the subsets containing perceptual coefficients (MERIP: 59.33% using MFCC, PESD: 61.5% using BFCC, PSSD: 80.68% using MFCC). In each case, these results are obtained using a different classification algorithms: k-NN, MLP, RF, for MERIP, PESD and PSSD respectively. The lowest results are collected in case of F0, formants and energy and this is noticeable for all datasets.

Analyzing results retrieved from different models, in most cases, a significant recognition rate improvement when using the RF classifier can be observed. it is very evident especially for MERIP and PSSD corpora, where the best results were gathered using RF for 6 out of 10 models in case of MERIP and 5 out of 10 in case of PSSD. When it comes to PESD, MLP gives the best recognition results for 4 out of 10 models. Other classifiers (k-NN, SMO, Bagging or LMT) give best results in individual cases, but without any repeatable pattern. SL, RC, NBTree and j48 algorithms did not take the lead in any model and thus will be omitted in further analysis.

There is a discrepancy between different types of databases (acted out: MERIP and PESD, and spontaneous PSSD) as well as between the same type of databases (MERIP and PESD). Thus, it can be assumed that recognition is affected not only by the type of database, but also by its size and by the type of samples such as uttered sentences and individual features of the speaker. Such varied results and the lack of repeatability indicates the necessity of conducting efficiency tests and selection of appropriate methods every time the corpora is modified.

### 4.2. Efficiency of Proposed Algorithm

**Figure 4:**
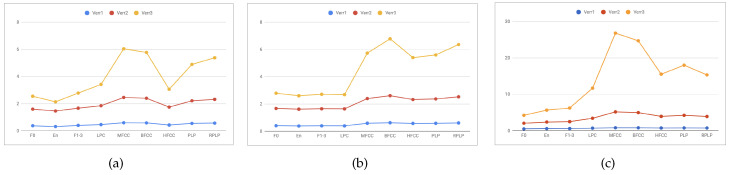
The discrete distribution of the error rate obtained by selected models for each features sets for (**a**) MERIP (**b**) PESD (**c**) PSSD.

Based on the results presented in the previous section, classifiers providing highest results on specific feature sets are selected to be part of the proposed algorithm. Thus, for example, in case of MERIP, the final algorithm consists of: RF for F0, LPC, HFCC, PLP, and RPLP LMT for energy and formants, k-NN for MFCC, MLP for BFCC. Next, the error rate of each model is taken into account to calculate the weights for weighted voting (see Figure 4). To assess the proposed method, the results are compared with those obtained using classical approach: using common classifiers on the whole feature set (see Table A1).

**Figure 5:**
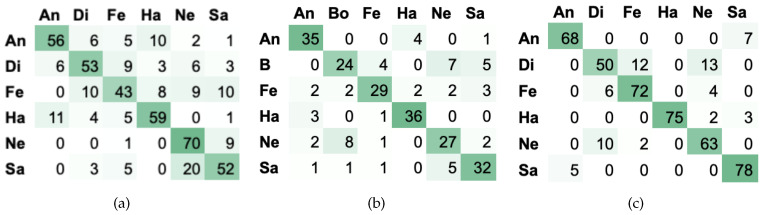
Confusion matrices presenting the best results obtained for (**a**) MERIP (**b**) PESD (**c**) PSSD. Emotional states: An—anger, Di—disgust, Fe—fear, Ha —happiness, Ne—neutral, Sa—sadness.

According to Table 5 and Table 6 an improvement of the overall accuracy using proposed algorithm can be observed in comparison to commonly used classifiers for all datasets. The lowest increase of results is observed for MERIP: MLP gives 66.9% and the third method of weighted voting 69.38%. It is important to note that equal voting among best models gives lower recognition than MLP. Significantly improved recognition quality can be observed in case of PESD and PSSD, where the proposed method boost the overall accuracy from 66.83% (k-NN) and 83.52% to 76.25% and 86.14% for weighted voting respectively. In case of PSSD dataset equal voting gives the same results as MLP. The average accuracy on MERIP, PESD and PSSD databases is illustrated as a confusion matrix in Figure 5.

The analysis of confusion matrix illustrates that the mistakes are different for each database. For example in case of MERIP anger and happiness are most confused and the same issue occurs for PESD. However, in case of PSSD misrecognition of anger and sadness is more clearly visible. Additionally, it can be observed that confusion between boredom-sadness-neutral is a common mistake for all datasets.

Table 7 presents the accuracy achieved in state-of-the-art research on PESD and PSSD datasets, which has been improved using the algorithm proposed in this paper. It is impossible to compare results for MERIP, since the database has been released recently and, up to now, there has been no research carried out on it.

In order to verify if acted out database can be used as a training set for application operating in real environment, selected classifiers were tested using mixed sets. In the first experiment, the training set consists of one of acted out databases (MERIP or PESD). In the second experiment both sets are connected, creating a larger training set. PSSD is a testing set in both cases. Obtained results are presented in Table 8.

As expected, the effectiveness of classifiers whose testing and training sets comprised different datasets is much lower in comparison to those operating on one particular database. When the acted out database is the training one, the average emotion recognition rate barely exceeds 30%. Increasing the number of samples in the training set by combining both acted out datasets, increased the quality of the classification. However, even in this case, the results do not exceed 50%. It should be noted that, as in previous cases, the proposed algorithm gives better results.

## 5. Conclusions

In this paper, performance of a committee of classifiers working on small subsets of features was studied and competitive performance in speech-based emotion recognition was shown. The proposed algorithm was tested on three different types of databases and in every case it achieved performance equal or better than current state-of-the-art methods. Although obtained results look promising when working within one particular database. When it comes to mixed database classification, the results are much lower and require further study. The research indicates that using the acted out database as a training set of a model that is supposed to operate in real conditions is not the perfect approach. To achieve higher results, it is recommended either to use a training set with bigger number of samples than a test set or train the model using spontaneous speech samples. This is crucial to create a system operating in real-world environment. Future works may include adding a gender recognition module right before emotional states classification, since a huge impact of gender on SER is noticed in many papers. It is also worth to explore and examine robust features, which have an impact on differentiation between emotional states with similar resonance such as anger and happiness, as well as neutral, sad and boredom. Additionally, replacing classic algorithm models with deep learning e.g., CNN or LSTM-RNN can be considered on the grounds that the use of neural networks provides good results in SER. At the same time, it must be emphasized that deep learning requires a large number of training samples whereas widely used and accessible databases still have their limitations.

## Figures and Tables

**Table 1:** Main characteristics of databases investigated in this research.

Database	No. of Samples/Per Emotion	Female/Male	Type	No. of Emotions
MERIP	560/unsp	8/8	acted	7: Ne, Sa, Su, Fe, Di, An, Ha
PESD	240/40	4/4	acted	6: Ha, Bo, Fe, An, Sa, Ne
PSSD	748/80	nd/nd	natural	8: Ha, Sa, An, Fe, Di, Su, An, Ne

**Table 2:** Average recognition results [%] of features subsets for MERIP database.

	k-NN	MLP	SL	SMO	Bagging	RC	j48	LMT	NBTree	RF
F0	34,02	32,39	34,75	34,75	34,75	34,75	30,26	33,33	26,47	**37,35**
En	30,91	29,03	26,93	25,99	33,49	28,57	26,22	**31,62**	29,03	28,1
F1-F3	36,06	35,36	39,11	36,06	34,43	33,02	29,03	**40,04**	33,26	33,3
LPC	42,39	45,2	44,73	41,22	39,11	42,39	32,55	44,26	34,43	**45,9**
MFCC	**59,33**	57,37	51,52	53,63	50,58	54,1	44,73	51,05	44,96	51,3
BFCC	57,21	**58,39**	52,69	54,56	51,05	53,63	45,19	57,61	46,37	56,9
HFCC	39,11	33,02	37	37	35,12	39,81	33,72	37,23	37	**42,86**
PLP	56,44	49,18	43,09	40,75	47,3	54,1	44,26	53,16	41,69	**54,8**
RPLP	55,5	43,09	39,11	43,32	50,11	52,46	46,37	47,54	40,28	**56,9**

**Table 3:** Average recognition results [%] of features subsets for PESD database.

	k-NN	MLP	SL	SMO	Bagging	RC	j48	LMT	NBTree	RF
F0	33,76	34,18	38,82	34,18	30,8	32,91	30,8	37,55	32,49	**40,08**
En	35,02	34,17	34,17	29,95	**37,97**	32,06	29,95	37,55	35,86	33,75
F1-F3	38,82	36,71	34,18	36,29	38,4	37,55	37,97	34,17	32,91	**39,24**
LPC	38,82	32,06	38,37	37,55	32,49	33,75	32,07	**38,96**	31,22	31,22
MFCC	38,82	**58,22**	51,89	57,38	45,99	49,36	41,77	51,89	39,24	54,43
BFCC	54	**61,6**	49,37	59,49	42,19	49,36	41,35	48,1	43,46	55,69
HFCC	55,27	51,05	56,19	**56,96**	47,26	50,21	42,62	55,27	41,77	52,74
PLP	**57,74**	53,58	56,96	49,36	47,68	54,85	44,72	55,69	43,04	56,11
RPLP	54,43	**60,34**	54,43	53,59	48,95	47,26	42,19	53,16	41,77	56,96

**Table 4:** Average recognition results [%] of features subsets for PSSD database.

Features:	k-NN	MLP	SL	SMO	Bagging	RC	j48	LMT	NBTree	RF
F0	48,86	48,57	50,28	48,01	**51,42**	49,14	41,76	51,13	42,04	53,69
En	55,39	52,84	45,45	42,61	**57,95**	50	50,85	55,11	51,7	55,96
F1-F3	53,97	52,56	57,95	**59,94**	54,26	53,41	50,28	57,67	45,45	58,52
LPC	65,9	**70,73**	67,89	66,19	61,93	66,19	63,92	67,89	57,38	68,18
MFCC	76,7	76,98	71,85	76,13	64,2	73,57	63,35	71,87	64,2	**80,68**
BFCC	76,32	72,29	77,55	77,55	69,66	71,36	62,85	77,7	63,46	**79,87**
HFCC	72,29	69,97	72,91	71,82	74,67	71,39	61,76	72,91	60,22	**74,61**
PLP	**76,42**	74,14	71,59	71,02	67,89	74,43	65,34	71,59	67,05	74,43
RPLP	72,29	69,45	68,11	69,67	66,09	71,21	55,72	68,11	54,48	**74,45**

**Table 5:** Average recognition results [%] of all features subsets for MERIP, PESD and PSSD database using commonly known classifiers.

	k-NN	MLP	SL	SMO	Bagging	RC	j48	LMT	NBTree	RF
MERIP	60,99	66,9	60,75	61,22	48,22	56,26	49,4	60,28	43,97	59,1
PESD	66,83	62,82	59,4	57,27	59,15	57	41,88	60,25	47,86	65,55
PSSD	78,97	83,52	80,96	83,23	74,43	77,84	65,9	80,68	69,03	81,53

**Table 6:** Average recognition results [%] of all features subsets for MERIP, PESD and PSSD database using proposed algorithm with equal voting (EV) juxtaposed with three different approaches for weighted voting.

	EV	$w_{i}=1-{\mathrm{err}}_{i}$	$w_{i}=\frac{1}{{\mathrm{err}}_{i}}$	$w_{i}={\left(\frac{1}{{\mathrm{err}}_{i}}\right)}^{2}$
MERIP	60,99	66.43	67.61	**69,38**
PESD	64,58	**76,25**	72,39	74,58
PSSD	83,52	84,94	85,22	**86,14**

**Table 7:** Comparison with similar works.

Database	Reference	Method	Accuracy
PESD	[65]	SVM	75,42
PESD	[66]	Binary Tree/SVM	56,25
PESD	[19]	EC (k-NN)	70,9
PSSD	[19]	EC (k-NN)	84,7
PSSD	[67]	k-NN/SVM	83,95

**Table 8:** The average emotion recognition rates for mixed database. Columns named EV, $Verr_{1}$, $Verr_{2}$, $Verr_{3}$ represent the voting methods proposed in this paper.

Training	Testing	k-NN	MLP	SL	Bagging	RC	RF	EV	${\mathrm{Verr}}_{1}$	${\mathrm{Verr}}_{2}$	${\mathrm{Verr}}_{3}$
MERIP	PSSD	31,94	29,94	32,84	30,67	29,03	30,12	31,65	32,91	33,02	33,26
PESD	PSSD	29,47	28,2	32,73	32	38,52	34,72	35,02	38,4	38,82	39,24
MERIP + PESD	PSSD	37,97	42,89	32,07	32,07	34,6	45,99	44,3	47,68	48,57	47,72

## Abbreviations

The following abbreviations are used in this manuscript:

BFCC	Bark Frequency Cepstral Coefficients
CNN	Convolutional Neural Network
EC	Ensemble Committee
EV	Equal Voting
HFCC	Human Factor Cepstral Coefficients
HMM	Hidden Markov Models
LSTM	Long Short-Term Memory
k-NN	k Nearest Neighbours
LDA	Linear Discriminant Analysis
LMT	Logistic Model Trees
MERIP	Multimodal Emotion Recognition in Polish
MFCC	Mel Frequency Cepstral Coefficients
MLP	Multilayer Perceptron
NBTree	Naive Bayes Tree
PCA	Principal Component Analysis
PESD	Polish Emotional Speech Database,
PLP	Perceptual Linear Prediction
PSSD	Polish Spontaneous Speech,
RC	Random Cometee
RF	Random Forest
RNN	Recurrent Neural Network
RPLP	Revised Perceptual Linear Prediction
SER	Speech Emotion Recognition
SMO	Sequential Minimal Optimization
SL	Simple Logistic
SVM	Support Vector Machines

## Appendix A

This section provides the details about selected features for each set.

**Table A1:** Feature selected applied on the whole sets for MERIP, PESD and PSSD corpora.

Database	Features
*MERIP*	F0: mean; energy: median, std; F1, F2: mean, median, F2, F3: max, min, F3:std, LPC mean: 2–11; MFCC mean: 1,6, 10, 11,12; MFCC std: 12, MFCC median: 6,11; MFCC min: 1; MFCC max: 7,11; BFCC mean: 1,6,10; BFCC median: 1,6; BFCC min: 1,6; BFCC max: 11; PLP mean: 5–8; PLP median: 6,10,11; PLP std: 3–10, RPLP mean: 1,3,5,9; RPLP median: 3; RPLP std: 5,9–10;
*PESD*	F0: min, upper quartile, variation rate, rising-ranege max; energy: min; F1-F2: mean; F3: median; F3: min; LPC mean: 2,3,7,8,11; LPC median: 2,7,8; LPC max: 7; MFCC mean: 3,4,6,7,12; MFCC std: 1,4,5,9; MFCC median: 4,6,8–11; MFCC min: 1,3–5; MFCC max: 4; BFCC mean: 1,4; BFCC median: 5–8; BFCC min: 4,6; BFCC max: 5–7; PLP max: 1; PLP min: 1–3,4,10; PLP std: 4–7,10; RPLP mean: 1,4,7,9; RPLP median: 4–7; RPLP std: 1,4;
*PSSD*	F0: mean, kurtosis; energy: median, std; F1:mean, median; F3: min, std; LPC mean: 5–7,10,11; MFCC mean: 1,3,9–11; MFCC std: 11,12, MFCC median: 1,7; MFCC min: 7; MFCC max: 1; BFCC mean: 1–4,10; BFCC median: 2,6,9; BFCC min: 1,5–8; BFCC max: 1–3; PLP mean: 5,6,10,12; PLP std: 3,7,10; PLP min: 1; PLP max: 8,9; RPLP mean: 1,3,5,9; RPLP median: 3; RPLP std: 5,9–10;

**Table A2:** Feature sets after subsets selection obtained for MERIP, PESD and PSSD corpora.

	*MERIP*	*PESD*	*PSSD*
*F0*	mean *F0*, median *F0*, std *F0*, max *F0*, range *F0*, upper quartile *F0*, lower quartile *F0*, interquartile range *F0*, skewness *F0*, kurtosis *F0*, *F0*, rising-slope max *F0*, falling-slope max *F0*;	mean *F0*, median *F0*, max *F0*, upper quartile *F0*, lower quartile *F0*, interquartile range *F0*, kurtosis *F0*, rising-range min *F0*, falling-range min *F0*;	mean *F0*, median *F0*, max *F0*, min *F0*, rane *F0*, upper quartile *F0*, lower quartile *F0*, interquartile range *F0*, skewness *F0*, falling-range max *F0*;
*F1-F3*	mean: *F1, F3*; max: *F1, F3*; median: *F1, F2, F3*, min: *F1, F3*; standard deviation *F1, F3*	mean: *F1, F3*; median: *F2, F3*, min: *F1*; standard deviation *F1, F3*;	mean: *F1, F2, F3*; max: *F1, F3*; median: *F1, F2*, min: *F1, F3*; standard deviation *F1, F2, F3*
*Energy*	max, min, median, std, range, mean	max, min, median, std	max, min, median, std, range
*LPC*	*LPC* mean: 2, 4–6,12	*LPC* mean: 5,7–11	*LPC* mean: 2–10
*MFCC*	*MFCC*: 1,3,6 -mean *MFCC*: 2,4,6 -median *MFCC*: 5,10 -std *MFCC*: 3,10 -max *MFCC*: 2,12 -min	*MFCC*: 1,3,9 -mean *MFCC*: 1 -median *MFCC*: 3,5,12,14 -std *MFCC*: 1,12 -max *MFCC*: 14 -min	*MFCC*: 2,6,7 -mean *MFCC*: 2 -median *MFCC*: 1,3,7 -std *MFCC*: 1,4 -max *MFCC*: 1,4 -min
*BFCC*	*BFCC*: 2,7,8 -mean *BFCC*: 7 - std *BFCC*: 1,2,3,11 -median *BFCC*: 1,4 -max *BFCC*: 2,5 -min	*BFCC*: 3,9 -mean *BFCC*: 4,10 -std *BFCC*: 2,3,5,7, -median *BFCC*: 1 -max *BFCC*: 2,3 -min	*BFCC*: 8 -mean *BFCC*: 1,2 -std *BFCC*: 2,6,12 -median *BFCC*: 1,2 -max *BFCC*: 4 -min
*HFCC*	*HFCC*: 1,2 -mean *HFCC*: 1,2,4,6 -std *HFCC*: 2,4 median *HFCC*: 1,7,10 -max *HFCC*: 5,6 -min	*HFCC*: 1,2,4 -mean *HFCC*: 1,3,4 -std *HFCC*: 1,4 median *HFCC*: 2,3 -max *HFCC*: 1,2 -min	*HFCC*: 3,5 -mean *HFCC*: 2,5,7 -std *HFCC*: 2,4 -max *HFCC*: 1,4,7 -min
*PLP*	*PLP*: 3,5 -mean *PLP*: 2,3,6 -median *PLP*: 7,10 -std *PLP*: 1,4,7 -max *PLP*: 1,4 -min	*PLP*: 1,5,9 -mean *PLP*: 4,6 -median *PLP*: 3,7 -std *PLP*: 1,2 -max *PLP*: 6 -min	*PLP*: 1 -mean *PLP*: 8,10 -median *PLP*: 1,4,6 -std *PLP*: 1,5 -max *PLP*: 1,9 -min
*RPLP*	*RPLP*: 1,2,3 -mean *RPLP*: 5,6,8 -median *RPLP*: 2 -std *RPLP*: 9–11 -max	*RPLP*: 1,12 -mean *RPLP*: 3–5 -median *RPLP*: 2,7 -std *RPLP*: 1–3,6, -max *RPLP*: 2,4 -min	*RPLP*: 2,3 -mean *RPLP*: 1 -median *RPLP*: 2,3 -std *RPLP*: 1,3,8 -max *RPLP*: 1,4 -min
